# Trends in adolescent first births in sub-Saharan Africa: a tale of increasing inequity?

**DOI:** 10.1186/s12939-020-01251-y

**Published:** 2020-09-04

**Authors:** Sarah Neal, Andrew Amos Channon, Venkatraman Chandra-Mouli, Nyovani Madise

**Affiliations:** 1 Department of Social Statistics and Demography, University of Southampton, Southampton, UK; 2 Department of Sexual and Reproductive Health and Research, World Health Organisation, Geneva, Switzerland; 3 African Institute for Policy Development (AFIDEP), Lilongwe, Malawi

**Keywords:** Adolescent pregnancy, Adolescent sexual health, Fertility, Sub-Saharan Africa, Equity

## Abstract

**Background:**

Single aggregate figures for adolescent pregnancy may fail to demonstrate particular population groups where rates are very high, or where progress has been slow. In addition, most indicators fail to separate younger from older adolescents. As there is some evidence that the disadvantages faced by adolescent mothers are greatest for those at the younger end of the spectrum, this is an important omission. This paper provides information on levels and trends of adolescent first births in 22 countries (at national and regional level) disaggregated by age (< 16 years, 16/17 years and 18/19 years), socio-economic status and place of residence. It highlights differences and similarities between countries in the characteristics of women who experience first birth during adolescence, as well as providing information on trends to identify groups where progress in reducing adolescent first births is poor.

**Methodology:**

In this descriptive and trend analysis study we used data from 22 low- and middle-income countries from sub-Saharan Africa that have at least three Demographic and Health Surveys (DHS) since 1990, with the most recent carried out after 2005. Adolescent first births from the most recent survey are analysed by age, wealth, and residence by country and region for women aged 20–24 years at time of survey. We also calculated annual percentage rates of change (using both short- and longer-term data) for adolescent first births disaggregated by age, family wealth and residence and examined changes in concentration indices (CI).

**Findings:**

Overall percentages of adolescent first births vary considerably between countries for all disaggregated age groups. The burden of first birth among adolescents is significant, including in the youngest age group: in some countries over 20% of women gave birth before 16 years of age (e.g. Mali and Niger). Adolescent first births are more common among women who are poorer, and live in rural areas; early adolescent first births before 16 years of age are particularly concentrated in these disadvantaged groups. Progress in reducing adolescent first births has also been particularly poor amongst these vulnerable groups, leading to increasing inequity.

**Conclusions:**

Findings from this study show that adolescent births are concentrated among vulnerable groups where progress is often poorest. Strategies and programmes need to be developed to reduce adolescent pregnancies in marginalised young women in low- and middle-income countries.

## Background

Each year, an estimated 16 million girls and young women give birth between the ages of 15 and 19 [1], and an additional one - two million give birth before their fifteenth birthdays [2, 3]. Most of these girls and young women live in low- or middle-income countries [1]. Reducing the proportion of young women who become mothers before the age of 20 years has been recognised as a key objective for the international development community by the inclusion of two targets to reduce adolescent fertility within the Sustainable Development Goals (SDGs) [4]. Adolescent fertility, particularly amongst the youngest age-group, carries significant health risks for both mother and infant [5–7], and limits education and livelihood opportunities for young women [8]. In sub-Saharan Africa is it also recognised that reducing adolescent pregnancy is an essential to achieving the demographic dividend [9], which offers unparalleled potential for sustained and accelerated economic growth within the region.

While sub-Saharan Africa countries reduced their adolescent fertility rate during the Millennium Development Goal era (1990–2015) from 140 to 101 births per 1000 women aged 15–19 years, this reduction is markedly less than other regions such as South Asia, North Africa and the Middle East [10].

Progress in reducing births in adolescents at national level is normally measured by either estimates of the percentage of women who have a first birth before the age of 20 years or the adolescent fertility rate (the number of births per thousand women aged 15–19 years). However, aggregate data of this type may conceal substantial subnational inequalities, especially when tracking progress over time. The level of adolescent fertility is underpinned by complex socio-economic, educational, cultural, geographic and service availability factors, with contexts, patterns and trends potentially varying markedly for different population groups within countries. The central commitment of the SDGs to “leave no-one behind” [11] requires that close attention is made to these differential patterns in order to enable programmes to be targeted at those most at risk of having an adolescent pregnancy and birth. It also enables approaches to be tailored for different populations, depending on the determinants or contexts of early pregnancy and child bearing within specific groups. Large-scale, nationally representative surveys such as Demographic and Health Surveys (DHS) and Multiple Indicator Cluster Surveys (MICS) provide opportunities for many countries to obtain more comprehensive information on adolescent fertility that incorporates some of the contextual, socio-economic and geographic factors.

This paper provides evidence on the trends in the levels of adolescent first birth in 22 East / Southern and Western / Middle African countries (at both national and regional levels) disaggregated by age, and in particular whether there is evidence of systematic poorer progress in particular population sub-groups. We set out two research questions:

- What progress has been made in reducing adolescent first births in sub-Saharan African countries, and how does progress in reducing adolescent first births differ across three adolescent age sub-categories (< 16 years, 16/17 years and 18/19 years), with a particular focus on the youngest age group?
- How has progress in reducing adolescent first births differed by socio-economic grouping and urban / rural residence, and is there evidence of changing patterns of inequalities between groups?

We particularly focused on first births because there is a danger that reductions in age-specific fertility (ASFR) rate for 15–19 year olds may mask a stagnation or even increase in the proportion of adolescents becoming mothers [12] if the reduction in ASFR is driven by reductions in subsequent births.

## Methodology

The study draws on data from 22 countries in sub-Saharan Africa that have at least three DHS since 1990, with the most recent carried out after 2005. DHS surveys (funded largely by the U.S. Agency for International Development) are nationally representative, large scale household surveys providing high-quality information on a range of indicators for a wide range of topics, including fertility and maternal health.

Based on the DHS regional classification, nine countries were in Western Africa, two were in Middle Africa, one was in Southern Africa and the remaining 10 were in Eastern Africa:

- Western Africa: Benin, Burkina Faso, Cote D’Ivoire, Ghana, Guinea, Mali, Niger, Nigeria, Senegal
- Middle Africa Cameroon, Chad
- East Africa: Ethiopia, Kenya, Madagascar, Malawi, Mozambique, Rwanda, Tanzania, Uganda, Zambia, Zimbabwe
- Southern Africa: Namibia

Due to small numbers, the two Middle African countries (Cameroon and Chad) were grouped with Western African countries and Namibia from the Southern African region was grouped with Eastern African countries for the purpose of aggregated regional analysis. These reflect UN regional groupings which often group Southern / Eastern and Western / Middle together [13, 14].

Country sample sizes of 20–24 year olds varied between 789 and 2844 in the baseline survey, with a mean average of 1565 for East / Southern Africa and 1287 for Western / Middle Africa. In the final surveys sample sizes ranged from 1720 to 6714, with a mean average of 2706 for Eastern / Southern Africa and 3118 for Western / Middle Africa. Full details of country sample sizes can be found in Additional file 1.

Adolescent first births from the most recent survey are analysed by age at first birth (disaggregated by < 16, 16/17 and 18/19 year age groups), household place of residence (urban/rural) and family wealth (measured through asset quintiles). This is conducted by country and further aggregated by regional groupings. In addition, trends in adolescent first births over time are also analysed using all three surveys (for < 16, 16/17 and 18/19 year age groups) disaggregated by place of residence and wealth quintile. Average annual percentage rates of change were calculated over both the short-term (average 8 years) and the medium-term (average 18 years). Confidence intervals for the proportion of adolescent births disaggregated for poorest / richest quintile and by urban / rural residence calculated using the logit function (which ensures that they remain between 0 and 1) can be found in Additional files 2 and 3.

All the analyses are based on a sample of women aged 20–24 years at time of survey. This ensures that all the samples are exposed to the full adolescent period of interest. There is also evidence that reporting of timing of sexual and reproductive health events by women younger than 20 years may be less reliable [15].

Changes in family wealth differentials are measured using ratios of the richest to poorest asset quintile (Q5:Q1), as well as concentration indices at regional level. The concentration index (CI) is defined as twice the overall area between the concentration curve and the line of equity, where the concentration curve shows the cumulative distribution of a health outcome against individuals ranked from poorest to richest (in this case based on the asset index). The CI ranges from − 1 to + 1, with an index of zero representing complete equality. Unlike ratio estimates of inequality the CI takes into account distribution for the whole range of wealth and not only in the richest and poorest quintiles. In this study, a positive CI indicates that the rich are more likely to have an adolescent birth, while a negative CI indicates the opposite. Sample weights were applied as necessary to account for differential chances of selection into the sample, but regional pooled data was not weighted by population size. Most analysis was carried out using STATA 14.0, but the CIs were calculated using Excel.

## Findings

### Overall trends

**Fig. 1:**
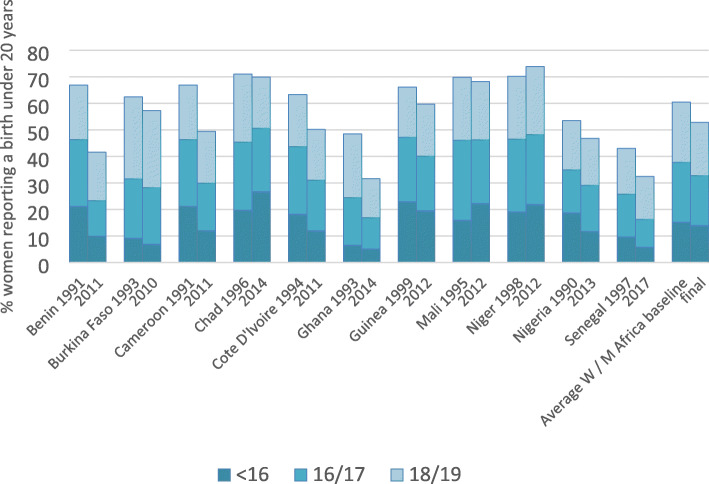
The percentage of women aged 20–24 who had a first birth before 20 years for 11 countries in West /Middle Africa disaggregated by age group (based on most recent and final DHS survey)

**Fig. 2:**
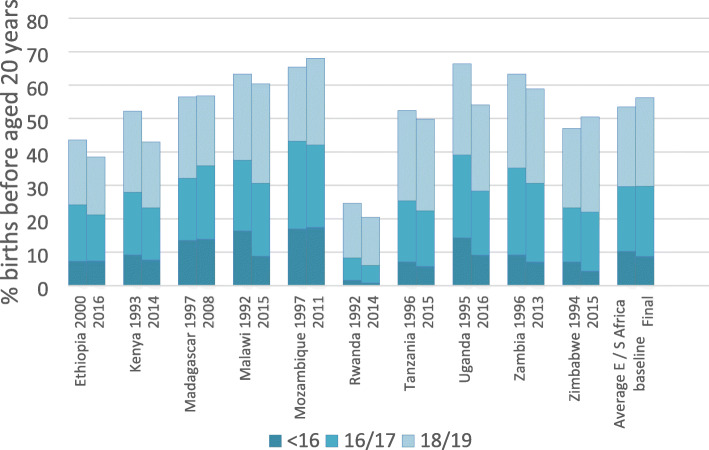
The percentage of women aged 20–24 who had a first birth before 20 years for 11 countries in East / Southern Africa disaggregated by age group (based on most recent and final DHS survey)

Table 1 and Figs. 1 and 2 show the percentage of women aged 20–24 who reported a first birth before the age of 20 years, disaggregated by age group for each country and for the regional groupings. The percentage of first births during the adolescent period is almost universally high, although there are stark differences between countries. In the most recent survey, women in Rwanda are the least likely to have given birth before 20 years, with 21% reporting a birth before this milestone. In contrast, in 12 countries, over 50% of women give birth before 20 years, with a maximum of 74% in Niger. Mean average percentages are similar for the two aggregate regional groupings, with an overall mean of 53% and 49% of first births before the age of 20 in Western/Middle and Eastern/Southern Africa respectively. However, the percentage of women reporting first births under the age of 16 years is markedly higher in Western/Middle Africa (14% as opposed to 8%). In Chad, Mali and Niger (which also have some of the highest rates of first births under 20 years) over 20% of women have their first birth before their 16th birthday, and 10% or more of women have had a first birth by this age in eight out of the 11 West/ Middle Africa countries.

**Table 1:** Estimates of the percentage of first births at ages < 16, 16/17, 18/19 and < 20 years for 22 sub-Saharan African countries for three consecutive DHS surveys (based on women aged 20–24 years at time of survey)

Country	Baseline Survey					Middle Survey					Final Survey (Most Recent)				
		< 16	16/17	18/19	< 20		< 16	16/17	18/19	< 20		< 16	16/17	18/19	< 20
Benin	1996	5.9	17.5	26.5	49.9	2006	8.8	14.4	21.9	45.1	2011	9.9	13.4	18.3	41.6
Burkina Faso	1993	9.2	22.3	30.9	62.4	2003	7.0	20.1	30.4	57.6	2010	6.9	21.3	29.1	57.3
Cameroon	1991	21.2	25.1	20.6	66.8	2004	14.2	18.9	22.1	55.2	2011	12.0	17.9	19.5	49.4
Chad	1996	19.7	25.7	25.6	71.0	2004	22.4	25.9	22.5	70.9	2014	26.7	23.9	19.3	69.9
Cote d’Ivoire	1994	18.2	25.5	19.6	63.2	1998	13.3	21.7	20.7	55.6	2011	12.0	19.1	19.1	50.2
Ghana	1993	6.5	18.0	24.0	48.5	2003	3.8	11.1	19.6	34.6	2014	5.1	11.8	14.7	31.6
Guinea	1999	22.9	24.3	18.9	66.2	2005	18.9	25.2	21.8	66.0	2012	19.6	20.5	19.6	59.6
Mali	1995	16.0	30.0	23.8	69.8	2006	19.8	26.5	23.2	69.4	2012	22.3	23.9	22.0	68.2
Niger	1998	19.1	27.4	23.7	70.2	2006	22.4	28.5	21.3	72.2	2012	21.9	26.3	25.6	73.9
Nigeria	1990	18.7	16.2	18.6	53.6	2008	12.5	15.2	15.2	42.9	2013	11.7	17.4	17.7	46.7
Senegal	1997	9.7	16.1	17.2	43.1	2010	9.3	12.3	15.9	37.4	2017	5.8	10.5	16.2	32.5
**Average W/M Africa**		15.2	22.6	22.7	60.4		13.9	20.0	21.3	55.2		14.0	18.7	20.1	52.8
Ethiopia	2000	7.3	16.9	19.4	43.6	2005	11.4	17	17.7	46.1	2016	7.4	13.8	17.3	38.4
Kenya	1993	9.2	18.7	24.3	52.1	2003	5.6	17.1	22.7	45.4	2014	7.7	15.6	19.7	43.0
Madagascar	1997	13.5	18.6	24.4	56.6	2003	9.8	21.5	22.5	53.8	2008	13.9	22.0	20.9	56.8
Malawi	1992	16.4	21.1	25.8	63.3	2004	10.9	23.2	29.0	63.2	2015	8.8	21.8	29.8	60.5
Mozambique	1997	17.0	26.2	22.2	65.3	2001	14.3	25.8	25.4	65.5	2011	17.4	24.7	25.9	68.0
Namibia	1992	4.6	13.2	23.8	41.6	2006	4.8	12.2	18.2	35.1	2013	3.3	11.6	19.8	34.6
Rwanda	1992	1.6	6.7	16.3	24.6	2005	1.0	6.7	14.6	22.3	2014	0.8	5.2	14.4	20.5
Tanzania	1996	7.1	18.3	27.0	52.4	2004	6.7	22.4	27.4	56.5	2015	5.7	16.7	27.4	49.8
Uganda	1995	14.3	24.8	27.3	66.4	2006	12.8	22.4	27.0	62.3	2016	9.1	19.2	25.8	54.1
Zambia	1996	9.2	26.0	28.1	63.3	2007	9.2	24.4	27.7	61.3	2013	7.1	23.5	28.3	58.9
Zimbabwe	1994	7.1	16.2	23.7	46.9	2005	4.5	16.2	26.1	46.9	2015	4.3	17.7	28.5	50.5
**Average S/ E Africa**		9.8	18.8	23.8	52.4		8.3	19.0	23.5	50.8		7.8	17.4	23.4	48.6

Trend analysis carried out over an average period of around 18 years shows limited change in any age group for most countries (Tables 1 and 2 and Additional file 4). Average annual percentage rates of change for Eastern/Southern and Western/Middle Africa for the medium term for births < 20 years are only − 0.3% and − 0.7% respectively. If we examine estimates of confidence intervals in Additional file 4, only five countries in Eastern/Southern Africa show a decline in the percentage of women experiencing a first birth under 20 years that cannot be attributed to sampling error at the 95% level (Kenya, Namibia, Rwanda, Uganda and Zambia). In Western/Middle Africa all countries except Niger, Chad and Mali show an often relatively modest but statistically significant (at the 5% level) decline. In Western/Middle Africa the fastest rate of progress has been made in the 16/17 year age group, whereas in Eastern/Southern Africa it is in the < 16 age group. If we compare the medium term with the more recent short term (average 8 years) there seems be a slower rate of change over the short compared to the medium term in all age groups within the Western/ Middle African region suggesting progress has slowed in recent years. In Eastern/Southern Africa there is a slightly greater rate of change in all age groups except < 16 years, suggesting progress in reducing adolescent first births has accelerated.

**Table 2:** Average annual percentage rate of change for adolescent first birth < 16, 16/17, 18/19 and < 20 years for 22 countries for women aged 20–24 years at time of survey based on DHS surveys: short and medium term

	medium term period in years	short term period in years	< 16 short	16/17 short	18/19 short	< 20 short	< 16 medium	16/17 medium	18/19 medium	< 20 medium
Benin	15	5	2.5	−1.4	−3.3	− 1.6	4.5	−1.6	−2.1	− 1.1
Burkina Faso	17	7	−0.2	0.9	− 0.6	− 0.1	− 1.5	− 0.3	− 0.3	− 0.5
Cameroon	20	7	−2.2	− 0.8	− 1.7	− 1.5	− 2.2	− 1.4	− 0.3	− 1.3
Chad	18	10	1.9	− 0.8	− 1.4	− 0.1	2.0	− 0.4	− 1.4	− 0.1
Cote d’Ivoire	17	13	− 0.8	− 0.9	− 0.6	− 0.7	− 2.0	− 1.5	− 0.2	− 1.2
Ghana	21	11	3.1	0.6	− 2.3	− 0.8	− 1.0	− 1.6	− 1.8	− 1.7
Guinea	13	7	0.5	−2.7	− 1.4	− 1.4	− 1.1	− 1.2	0.3	− 0.8
Mali	17	6	2.1	− 1.6	− 0.9	− 0.3	2.3	−1.2	− 0.4	− 0.1
Niger	14	6	−0.4	−1.3	3.4	0.4	1.0	−0.3	0.6	0.4
Nigeria	23	5	−1.3	2.9	3.3	1.8	−1.6	0.3	−0.2	−0.6
Senegal	20	7	−5.4	−2.1	0.3	−1.9	−2.0	− 1.7	−0.3	−1.2
**Average W / M Africa**	**17.7**	**7.6**	0.0	−0.8	− 0.6	− 0.5	−0.5	− 0.9	−0.7	− 0.7
Ethiopia	16	11	−3.2	−1.7	−0.2	−1.5	0.1	−1.1	−0.7	−0.7
Kenya	21	11	3.4	−0.8	−1.2	−0.5	− 0.8	−0.8	− 0.9	−0.8
Madagascar	11	5	8.4	0.5	−1.4	1.1	0.3	1.7	−1.3	0.0
Malawi	23	11	−1.8	−0.5	0.3	−0.4	−2.0	0.1	0.7	−0.2
Mozambique	14	10	2.2	−0.4	0.2	0.4	0.2	−0.4	1.2	0.3
Namibia	21	7	−4.5	−0.7	1.3	−0.2	−1.3	− 0.6	−0.8	− 0.8
Rwanda	22	9	−2.2	−2.5	−0.2	−0.9	− 2.3	−1.0	−0.5	− 0.8
Tanzania	19	11	−1.4	−2.3	0.0	−1.1	−1.0	−0.5	0.1	−0.3
Uganda	21	10	−2.9	−1.4	−0.4	−1.3	− 1.7	−1.1	− 0.3	−0.9
Zambia	17	6	−3.8	−0.6	0.4	−0.7	−1.3	−0.6	0.0	−0.4
Zimbabwe	21	10	−0.4	0.9	0.9	0.8	−1.9	0.4	1.0	0.4
**Average E / S Africa**	**18.7**	**9.2**	−0.5	−0.9	−0.1	− 0.5	−1.1	−0.4	0.0	−0.3

**Fig. 3:**
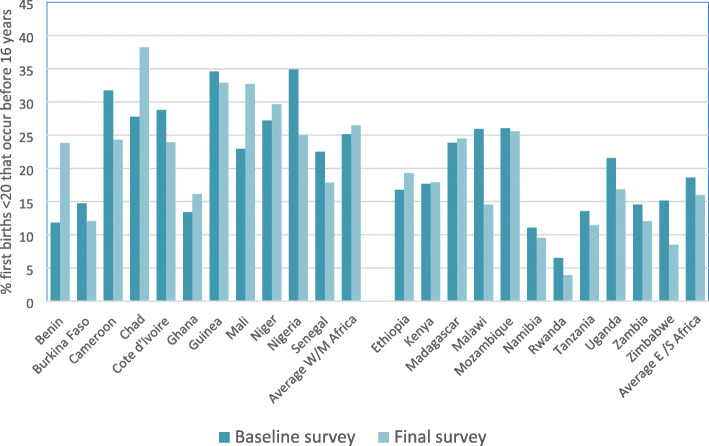
Percentage of all first births under the age of 20 years reported as below 16 years for women aged 20–24 at time of survey (based on baseline and final surveys)

Some countries have had large declines in the percentage giving birth under the age of 16: Cameroon, Cote d’Ivoire, Senegal, Malawi and Rwanda are notable in this regard with a reduction over the medium term of 2% or more per annum. These countries, in addition to Zimbabwe, Ghana and Nigeria, show declines that cannot be attributed to sampling error at the 95% level. Zambia, Ethiopia and Namibia also show positive signs of progress in the short term. However, there is a marked and significant *increase* in Benin, Chad and Niger: the increase is particularly marked within Benin, where < 16 first births have almost doubled from 6 to 10%. It is worth noting that differential patterns of progress between age groups have resulted in a fall in the proportion of all first births under 20 years that occur in the < 16 age group in Eastern/Southern Africa (from 19 to 16%), but this percentage has risen very slightly in Western/Middle Africa from 25 to 26%, with some countries (e.g. Benin, Chad and Mali) showing very large increases (see Fig. 3). A number of other countries show more modest increases or stagnation (e.g. Niger, Ethiopia, and Mozambique).

#### Trends by population sub-group

Tables 3 and 4 shows trends over time for women in the poorest and richest quintiles (see Additional file 3 for confidence intervals). Unsurprisingly there is a strong wealth gradient for first births under 20 years, and this is particularly strong for the youngest age group (< 16 years). For the richest group, there was a fall in the percentage of first births during the adolescent period in all countries, while at the regional level this reduction was seen for each different age at birth groups. However the poorest quintile suggested a very different pattern: Overall the percentage of women who gave birth < 20 years increased from 61 to 66%% in Eastern/Southern Africa, and remained static at 71% in Western/Middle Africa with apparent increases in eight Eastern/Southern and five Western/Middle African Countries.

**Table 3:** Baseline and final survey estimates for adolescent first birth for poorest and richest quintile disaggregated by age groups for women aged 20–24 years at time of survey for 22 countries based on DHS data

	Baseline survey: poorest quintile				Baseline survey: richest quintile				Final survey: poorest quintile				Final survey: richest quintile			
	< 16	16/17	18/19	< 20	< 16	16/17	18/19	< 20	< 16	16/17	18/19	< 20	< 16	16/17	18/19	< 20
Ethiopia	11.7	17.1	15.1	43.9	4.5	11.2	14.8	30.5	11	20.1	26.3	57.3	2.8	4.9	9.2	16.9
Kenya	14.8	24.1	26.5	65.3	5.5	13.9	18.1	37.6	14.6	22.9	28.4	65.8	3.6	8.7	13	25.2
Madagascar	24.1	21.8	31.1	77	5.8	6.2	19	31	29.5	26.4	22.5	78.4	2.6	14.1	16.1	32.8
Malawi	19.7	18.3	24	62.1	9.4	18.6	23	51	11.1	26.8	34.2	72	5.9	13.8	18.9	38.6
Mozambique	22.4	29.7	16.5	68.5	6.8	27.9	25.5	60.1	24.8	25.6	26.6	77	6.5	22.7	24.2	53.4
Namibia	7.8	14.6	22.7	45.1	1.7	11.1	21.8	34.6	6.1	20.5	30.1	56.7	0.2	5.7	11.6	17.4
Rwanda	1.6	8.7	23.5	33.8	0.6	4.6	9	14.1	0.9	8.9	22	31.7	1.2	3.9	9.2	14.3
Tanzania	10.9	27.4	24.6	62.9	4.5	14.6	22.3	41.4	7.7	28.1	33.4	69.1	3.7	6	16	25.7
Uganda	17.2	26.4	30.8	74.4	8.5	18.7	26.3	53.5	14.7	28.3	29.9	72.9	5.6	10.4	20.2	36.2
Zambia	11.8	29.1	33.7	74.6	5.5	19.3	17.7	42.6	9.3	33.3	33.4	76	5	9.8	19.5	34.3
Zimbabwe	12.8	20.2	31.5	64.4	2.9	10.7	17.1	30.7	7.6	26.4	39.7	73.8	0.7	5.5	17.4	23.6
**Average E/S Africa**	**14.1**	**21.6**	**25.5**	**61.1**	**5.1**	**14.3**	**19.5**	**38.8**	**12.5**	**24.3**	**29.7**	**66.4**	**3.4**	**9.6**	**15.9**	**28.9**
Benin	10.3	19.6	32.6	62.5	0.3	10.4	13	23.7	15.7	18.9	24.1	58.8	2.6	5.2	11.8	19.7
Burkina Faso	9.7	23.4	36.8	69.8	5.9	17.3	24.3	47.5	12.5	29.7	29	71.2	3	11.6	17.8	32.4
Cameroon	33.1	24.6	19.1	76.8	8.7	22.5	17.9	49.1	24.3	27.8	22.1	74.2	3.5	8.9	13.3	25.7
Chad	19.3	27	27.3	73.6	17.9	24.1	25.4	67.4	28.4	26.7	23.4	78.5	17.6	17.2	17.5	52.3
Cote d’Ivoire	25.2	35.1	16.7	77	10.3	15.9	15.6	41.9	18.5	29.4	22.8	70.8	5.2	10.1	11.7	27
Ghana	9.1	15.5	28.2	52.7	3.2	10.1	15.9	29.1	7.9	15.3	22.8	45.9	2.5	5.9	5.4	13.9
Guinea	33.8	27.6	16.3	77.7	11.7	20	12.3	44	26.2	23.3	28.1	77.6	8.4	13.8	14.5	36.7
Mali	15.7	33.6	25.7	75	11.2	19.7	21.1	52	26.4	28.3	22.6	77.3	12.3	18.4	17.3	47.9
Niger	19.8	37	23.6	80.5	10.6	17.1	21.5	49.2	22.6	31.9	26.5	80.9	11.5	21.2	20.3	52.9
Nigeria	25.7	23	22.1	70.8	8.0	10.3	14.6	32.9	26.1	30.9	19.7	76.7	3.1	5.7	9.5	18.3
Senegal	16.1	26.2	20.9	63.2	3.1	8.1	6.8	18	16.4	24.6	23.2	64.1	0.8	2.6	10.3	13.7
**Average W/M Africa**	**19.8**	**26.6**	**24.5**	**70.9**	**8.3**	**16.0**	**17.1**	**41.3**	**20.5**	**26.1**	**24.0**	**70.5**	**6.4**	**11.0**	**13.6**	**31.0**

**Table 4:** Ratio of richest to poorest quintile and concentration indices for adolescent first births disaggregated by age: based on DHS data from 22 countries for women aged 20–24

Years at time of survey				
	< 16	16/17	18/19	< 20
**Ratio richest**: **poorest quintile**				
**W/M Africa**				
Baseline survey	0.42	0.60	0.70	0.58
Final survey	0.31	0.42	0.57	0.44
**E/S Africa**				
Baseline survey	0.36	0.66	0.77	0.64
Final survey	0.28	0.39	0.54	0.44
**Concentration indices**				
**W/M Africa**				
Baseline survey	−0.18	−0.11	−0.09	−0.12
Final survey	−0.27	−0.13	−0.12	−0.16
**E/S Africa**				
Baseline survey	−0.19	−0.10	−0.08	−0.84
Final survey	−0.24	−0.17	−0.14	−0.15

The percentages of first births to mothers under the age of 16 years suggests a reduction for the richest quintile in both regions. It has reduced in the poorest in Eastern/Southern Africa, but has actually increased very slightly in this group for Western/Middle Africa. While some countries displayed marked improvements in reducing first births in < 16 year olds among the poorest (Cameroon, Cote D’Ivoire, Malawi and Zimbabwe) others showed very marked increases (e.g. Benin, Madagascar, Mali and Chad). These individual country level statistics need to be interpreted with caution due to relatively small sample sizes. However, in some countries e.g. Mali and Chad, the increase is extremely marked (the percentage of first births < 16 years in Chad in the poorest quintile has increased by over 10 percentage points).

The overall lack of progress among the poorest quintile has led to an increase in the ratio of richest to poorest quintile (Q5:Q1) for all age groups in both regions, as well as changes in the concentration indices that also demonstrate increasing inequality for all age groups of births (see Table 4).

Rates of first births are higher in rural than in urban areas for all countries in all age groups, and at the aggregate regional level urban / rural differentials are greatest for the < 16 age group for both Eastern/Southern and Western/Middle Africa (see Table 5). If we examine trends over time, on average rates of first births have dropped for all age groups in both regions for urban areas, and all countries have shown an overall fall in urban births under 20 years: in particular the fall in Western/Middle Africa in urban areas is very marked. However, if we examine rural areas, overall Eastern/Southern Africa demonstrates a slight increase in the percentage of first births under 20 years, and Western/Middle Africa showed a much smaller decrease than in urban areas. Overall the urban / rural ratio has increased for all age groups in both Eastern/Southern and Western/Middle Africa indicating increasing inequality.

**Table 5:** Baseline and final survey estimates for adolescent first birth for urban and rural for women aged 20–24 years at time of survey disaggregated by age groups

	Urban Baseline				Rural Baseline				Urban Final			Rural Final				
	< 16	16/17	18/19	< 20	< 16	16/17	18/19	< 20	< 16	16/17	18/19	< 20	< 16	16/17	18/19	< 20
**Benin**	3.1	15.6	19.4	38.1	7.8	18.8	31.3	57.9	6.2	10	15.3	31.5	13.6	16.8	21.2	51.6
**Burkina Faso**	5.5	19.7	21	46.2	10.3	23.0	33.6	66.9	2.6	11.5	18.8	32.9	8.8	25.7	33.7	68.2
**Cameroon**	17.5	21.9	20	59.4	24.3	27.7	21.1	73.1	7.8	13.6	16.9	38.3	18.2	24.1	23.3	65.6
**Chad**	16.4	25.8	25.5	67.7	20.8	25.7	25.7	72.1	19.6	17.3	17	53.9	29.3	26.4	20.2	76.0
**Cote d’Ivoire**	13.6	20.5	19.3	53.4	22.1	29.9	19.9	71.9	8.4	12	15.3	35.7	16.3	27.7	23.7	67.7
**Ghana**	4.9	13.5	18.5	36.9	7.5	20.8	20.4	55.9	4.2	8.7	9.6	22.4	6.1	15.4	20.6	42.1
**Guinea**	13.3	19.7	16.4	49.4	28.6	27.1	20.4	76.1	10.9	14.8	16.9	42.6	26.3	24.9	21.7	72.9
**Mali**	11.8	22.2	23.7	57.7	18.2	34.1	23.9	76.2	14.2	19.7	18.5	52.4	25.4	25.5	23.4	74.3
**Niger**	10	17.5	22.1	49.6	21.7	30.4	24.2	76.3	10.6	15.6	22.9	49.1	24.7	29.1	26.4	80.2
**Nigeria**	9.3	12	17	38.3	22.3	17.8	19.3	59.4	5.3	9.4	13.4	28.1	16.2	22.9	20.7	59.8
**Senegal**	4.8	10.5	12.6	27.9	13.8	20.8	21	55.6	2.7	5.8	11.2	19.7	8.8	15.1	21	44.8
**W/M Regional Average**	**10.0**	**17.9**	**19.6**	**47.5**	**17.5**	**25.1**	**25.0**	**67.5**	**8.4**	**12.6**	**16.0**	**37.0**	**17.6**	**23.1**	**23.3**	**63.9**
***Urban/ rural ratio***					***0.56***	***0.72***	***0.80***	***0.71***					***0.48***	***0.55***	***0.69***	***0.58***
**Ethiopia**	5.6	11.5	11.3	28.5	7.7	18.1	21.3	47.1	1.1	5	6.4	12.5	9.4	16.5	20.7	46.6
**Kenya**	4.5	16.2	18.5	39.2	10.6	19.5	26	56.1	6.1	11.7	17	34.7	9.2	19.3	22.2	50.8
**Madagascar**	8.5	13.8	21.9	44.2	15.5	20.5	25.4	61.4	4.2	14.3	19.4	37.9	16.1	23.7	21.3	61.1
**Malawi**	11.2	21.8	23.3	56.3	17.3	21	26.3	64.6	6.5	15	20.6	42.1	9.3	23.4	32	64.7
**Mozambique**	10.9	29.2	25.2	65.3	19	25.2	21.2	65.4	11.1	23.8	23.6	58.5	21.4	25.2	27.3	73.9
**Namibia**	2.8	15.5	25.2	43.4	5.8	11.8	22.9	40.4	2.5	9.6	16.8	28.9	4.5	14.9	24.7	44.1
**Rwanda**	2.0	5.7	11.7	19.4	1.6	6.8	16.7	25.1	1.3	5.3	13	19.5	0.7	5.2	14.8	20.7
**Tanzania**	5.5	15.9	25.7	47.1	7.7	19.2	27.6	54.5	4.7	10.8	19.5	35	6.4	20.3	32.2	58.9
**Uganda**	7.5	17.2	28.6	53.3	15.6	26.3	27.1	69	5.6	12.6	20.8	39	10.7	22	27.9	60.6
**Zambia**	8	24.1	24.1	56.2	10.2	27.6	31.3	69.1	5.8	16.2	25.1	47.1	8.5	30.9	31.5	70.9
**Zimbabwe**	3.2	12	19.5	34.7	9.4	18.7	26.1	54.2	2.3	11.9	21	35.3	5.7	22.1	34.2	62
**E / S Regional Average**	**6.3**	**16.6**	**22.0**	**44.9**	**10.9**	**19.5**	**23.3**	**53.8**	**4.7**	**12.4**	**18.5**	**35.5**	**9.3**	**20.3**	**26.3**	**55.8**
***Urban / rural ratio***					***0.58***	***0.85***	***0.94***	***0.84***					***0.50***	***0.61***	***0.70***	***0.64***

## Discussion

This analysis suggests limited and patchy progress has been made in reducing adolescent first births in sub-Saharan Africa over the last two decades. Eastern and Southern Africa has shown slightly faster progress in reducing adolescent pregnancy in the short term, but the situation is reversed for Western and Middle Africa, and there is no evidence of widespread accelerated progress in more recent years. In many countries a large proportion of women have their first baby before the age of 20 years, and in many countries, particularly in Western/Middle Africa a notable proportion of this group have given birth before the age of 16 years. However, within regions there is considerable heterogeneity (although very few countries have made marked progress in reducing adolescent first births over the time period studied).

Heterogeneity of progress in reducing adolescent first births is somewhat difficult to explain. Levels of adolescent motherhood are associated with marriage, female education and economic progress, as well as cultural factors and access to family planning, but these factors are often complex and inter-related. While early marriage is declining somewhat in sub-Saharan Africa, it remains prevalent in many countries and progress is inconsistent [16], which may partially explain persisting high levels of adolescent first births. Level of education is recognised as an important determinant [17], but the relationship at national level between educational attainment and adolescent pregnancy is not straightforward. While access to primary education for girls has increased markedly in most countries within sub-Saharan Africa over the last three decades [18], this is often not reflected in positive changes in adolescent first births, which may reflect the persistence of poor access to secondary schooling. Further research is needed to analyse more precisely the relationship between education and adolescent motherhood in terms of level and quality of schooling, as well as how this is affected by other socio-economic and cultural factors, and health care availability.

It might have been hoped that even if the reduction in adolescent first births has not been marked, the proportion occurring among the youngest, most vulnerable group would have reduced. However, in many countries our analysis suggests the opposite. The marked rise in very early adolescent births seen in Mali, Chad and Benin are particularly concerning, and the fact that these increases have occurred during a period of modest decline in other age groups suggests different pathways and drivers. The fact that in the Western/Middle Africa region the proportion of first births < 20 years occurring in girls under 16 years has actually increased is also concerning, and points to the need for further study on the underlying specific factors for this age group. It is also worth noting that in many countries reductions in marriage for those under 15 years has been differentially poor compared to reductions for women aged 15–17 years [16]. These very young adolescents are disadvantaged on a number of levels. There is evidence that adolescent mothers under the age of 16 years suffer from greater health risks to both themselves and their babies than older adolescents [5, 19–22]. In addition, the < 16 years group are particularly concentrated amongst the poorest and rural residents, who have less access to reproductive and sexual health information and services. It is vital that programmes aimed at reducing adolescent pregnancy focus on these younger adolescents, who may be restricted from accessing other initiatives or services due to cognitive, financial or logistic limitations. Research on what approaches are most successful for this age group is limited, but studies suggest a more holistic approach focused on individual and social assets may be more appropriate [23]. It is also important to note that among this younger age group sex is more likely to be coerced, highlighting the important links between sexual health and child protection for this vulnerable group [24].

Rising levels of very early adolescent motherhood (< 16 years) in Chad, Mali and Niger could partly be explained by the presence of armed conflict and instability during the period. Findings from a recent systematic review suggested that the increase in early marriage and subsequent childbearing that may occur as a result of armed conflict may be particularly focussed amongst the youngest adolescents [25] and indeed there is evidence that there has been an increase in marriage in very young adolescents in Mali within conflict-affected communities [26]. This often reflects the desire for families to “protect” their daughters from risk of sexual violence or perceived threats to moral welfare resulting from the collapse of community and social structure and mores, or from financial considerations [27]. In these countries the vast majority of women who give birth before 16 years are married at the time of birth so this trend may well follow increases in early marriage. However, Benin has been relatively politically stable in the last few decades. In this context, there is strong evidence of a high risk of sexual assault and violence against adolescent girls, particularly in the school environment, which may be partially driving these figures [28]. Further studies are needed to better understand the context of very early adolescent pregnancy in countries where it is increasing.

Our findings also clearly point to sharp socioeconomic and urban / rural inequities in adolescent first births, which appear to be increasing over time. This apparent concentration of adolescent births among the poorest and those in rural areas has important implications for the SDG commitment to “leave no-one behind” and highlights the need for nuanced indicators that capture these increasing inequalities. Evidence of growing inequity has important implications for programme development, as the poorest and those in rural areas are often inadequately served by large-scale programmes to improve adolescent access to contraception, as for instance they may be out of school or less able to access media-based campaigns [29]. More broadly, adolescents face significant barriers to SRH services, and in particular unmarried adolescents experience stigma and discrimination from both communities and health care providers [30]. Efforts to promote an enabling environment that supports adolescent SRH and rights are likely to require widespread changes and interventions [31] However, it must also be recognised that poverty, lack of education and the normalization of sexual abuse underpin inequities in adolescent childbearing [29]. The concentration of births within the youngest age groups in the poorest quintile points to a compounding of vulnerability. Given this, multisectoral approaches that address these structural drivers are key.

This paper has a number of limitations, which should be highlighted. Firstly it relies on recall data from survey respondents, and there is strong evidence that the reporting of sexual and reproductive health events may be prone to both intentional and unintentional bias [15, 32, 33]. Some studies indicate a tendency for very young adolescents to overstate their age at time of survey (and therefore overstate their age at first birth), and this risk has been reduced by using the 20–24 year cohort.

Further issues are that measures of wealth and place of residence are taken at time of survey rather than at the time of adolescent birth. Therefore it is impossible to clearly state whether these factors are determinants of adolescent birth, or outcomes: for instance adolescent motherhood may result in poor long term socio-economic prospects or migration. Longitudinal studies are needed to examine these issues more thoroughly. Sample sizes for some countries, particularly for the disaggregated groups, may also be relatively small, leading to large confidence intervals which means findings should be interpreted with some caution (see Additional files 2 and 3). In addition, we do not calculate and analyse wealth quintiles separately by urban and rural residence: the majority of those in the lowest quintiles will be rural, and it is not possible to identify whether there is poor progress among the urban poor. Further work is currently ongoing to explore this issue.

The heterogeneity of progress within the geographic groups points to the value of using aggregate data with caution. Further research is needed to consider what characteristics underpin the different patterns of adolescent motherhood, and whether a more useful typology can be developed that group countries in a manner that aids understanding of how best intervention programmes can respond. Careful analysis at the individual country level is also vital in understanding trends and patterns specific to that context.

## Conclusion

Disaggregating data on trends in adolescent first birth suggests that there is little progress in many sub-Saharan African countries. Where there is progress, it is heterogeneous, with little consistency as to which of the age categories has made most progress. However, in a number of countries (particularly in Western/Middle Africa) progress in reducing births amongst women younger than 16 years has been slower than for older ages, resulting in a greater proportion of adolescent first births occurring in this youngest age group. This is concerning as there are increased risk of poor outcomes for both mother and infant in this age range. There is a very strong wealth gradient, and markedly higher proportions of adolescent births in rural areas in all countries studied. Differentially poor progress amongst the poorest and rural residents has lead to adolescent mothers becoming increasingly concentrated among these groups over time. Younger adolescent births are disproportionately likely to be poor and rural, meaning their disadvantage is further compounded. More detailed and nuanced data on trends disaggregated by age, wealth and place of residence is vital for identifying vulnerable groups and targeting resources effectively.

## Supplementary information

**Additional file 1.** Sample size for all surveys: women aged 20–24 years.

**Additional file 2.** Confidence intervals of proportion of women experiencing a first birth < 16, 16/17, 18/19 and < 20 year by urban / rural residence.

**Additional file 3.** Confidence intervals of proportion of women experiencing a first birth < 16, 16/17, 18/19 and < 20 year by richest / poorest wealth quintile.

**Additional file 4.** Estimates of % of women aged 20–24 who had first birth at < 16, 16/17, 18/19 and < 20 years for baseline and final surveys with 95% confidence intervals.

## Data Availability

Data are publicly available from https://dhsprogram.com/

## Abbreviations

CI: Concentration index
DHS: Demographic and Health Survey
MICS: Multiple Indicator Cluster Survey
SDGs: Sustainable Development Goals
