# Supplementary material for: Trends in adolescent first births in sub-Saharan Africa: a tale of increasing inequity?
Source: Int J Equity Health. 2020 Sep 4;19:151. doi: 10.1186/s12939-020-01251-y (PMC7487507; doi:10.1186/s12939-020-01251-y)
Supplement: Supplementary file 4 — Additional file 4. Estimates of % of women aged 20–24 who had first birth at < 16, 16/17, 18/19 and < 20 years for baseline and final surveys with 95% confidence intervals. [file 12939_2020_1251_MOESM4_ESM.docx]

Appendix 1: Estimates of % of women aged 20-24 who had first birth at <16, 16/17, 18/19 and <20 years for baseline and final surveys with 95%confidence intervals

| **Country** | **Year** | **Estimate <16 years** | **Lower CI** | **Upper CI** | **Year** | **Estimate <16 years** | **Lower CI** | **Upper CI** |
| --- | --- | --- | --- | --- | --- | --- | --- | --- |
| **Ethiopia** | 2000 | 7.3 | 6.4 | 8.3 | 2016 | 7.4 | 5.8 | 9.0 |
| **Kenya** | 1993 | 9.2 | 7.8 | 10.6 | 2014 | 7.7 | 6.8 | 8.6 |
| **Madagascar** | 1997 | 13.5 | 11.7 | 15.4 | 2008 | 13.9 | 12.6 | 15.1 |
| **Malawi** | 1992 | 16.4 | 14.1 | 18.7 | 2015 | 8.8 | 7.9 | 9.7 |
| **Mozambique** | 1997 | 17.0 | 15.2 | 18.7 | 2011 | 17.4 | 15.9 | 18.9 |
| **Namibia** | 1992 | 4.6 | 3.3 | 5.8 | 2013 | 3.3 | 2.4 | 4.1 |
| **Rwanda** | 1992 | 1.6 | 0.9 | 2.3 | 2014 | 0.8 | 0.4 | 1.2 |
| **Tanzania** | 1996 | 7.1 | 5.8 | 8.3 | 2015 | 5.7 | 4.6 | 6.8 |
| **Uganda** | 1995 | 14.3 | 12.5 | 16.0 | 2016 | 9.1 | 8.1 | 10.2 |
| **Zambia** | 1996 | 9.2 | 7.9 | 10.5 | 2013 | 7.1 | 6.2 | 8.1 |
| **Zimbabwe** | 1994 | 7.1 | 5.6 | 8.5 | 2015 | 4.3 | 3.1 | 5.4 |
| **Country** | **Year** | **Estimate 16/17 years** | **Lower CI** | **Upper CI** | **Year** | **Estimate 16 / 17 years** | **Lower CI** | **Upper CI** |
| **Ethiopia** | 2000 | 16.9 | 15.5 | 18.3 | 2016 | 13.8 | 11.8 | 15.7 |
| **Kenya** | 1993 | 18.7 | 16.8 | 20.6 | 2014 | 15.6 | 14.3 | 16.9 |
| **Madagascar** | 1997 | 18.6 | 16.5 | 20.7 | 2008 | 22.0 | 20.5 | 23.5 |
| **Malawi** | 1992 | 21.1 | 18.6 | 23.7 | 2015 | 21.8 | 20.4 | 23.2 |
| **Mozambique** | 1997 | 26.2 | 24.1 | 28.3 | 2011 | 24.7 | 23.0 | 26.4 |
| **Namibia** | 1992 | 13.2 | 11.2 | 15.3 | 2013 | 11.6 | 9.9 | 13.3 |
| **Rwanda** | 1992 | 6.7 | 5.3 | 8.1 | 2014 | 5.2 | 4.3 | 6.1 |
| **Tanzania** | 1996 | 18.3 | 16.4 | 20.1 | 2015 | 16.7 | 14.9 | 18.4 |
| **Uganda** | 1995 | 24.8 | 22.7 | 27.0 | 2016 | 19.2 | 17.8 | 20.6 |
| **Zambia** | 1996 | 26.0 | 24.0 | 28.1 | 2013 | 23.5 | 22.0 | 25.0 |
| **Zimbabwe** | 1994 | 16.2 | 14.2 | 18.3 | 2015 | 17.7 | 15.6 | 19.9 |
|  |  |  |  |  |  |  |  |  |
| **Country** | **Year** | **Estimate 18/19 years** | **Lower CI** | **Upper CI** | **Year** | **Estimate 18/19 years** | **Lower CI** | **Upper CI** |
| **Ethiopia** | 2000 | 19.4 | 17.9 | 20.8 | 2016 | 17.3 | 15.2 | 19.4 |
| **Kenya** | 1993 | 24.3 | 22.2 | 26.4 | 2014 | 19.7 | 18.3 | 21.1 |
| **Madagascar** | 1997 | 24.4 | 22.1 | 26.7 | 2008 | 20.9 | 19.5 | 22.4 |
| **Malawi** | 1992 | 25.8 | 23.1 | 28.6 | 2015 | 29.8 | 28.2 | 31.4 |
| **Mozambique** | 1997 | 22.2 | 20.2 | 24.1 | 2011 | 25.9 | 24.1 | 27.6 |
| **Namibia** | 1992 | 23.8 | 21.2 | 26.3 | 2013 | 19.8 | 17.6 | 21.9 |
| **Rwanda** | 1992 | 16.3 | 14.3 | 18.4 | 2014 | 14.4 | 11.9 | 14.4 |
| **Tanzania** | 1996 | 27.0 | 24.9 | 29.2 | 2010 | 27.4 | 25.5 | 29.5 |
| **Uganda** | 1995 | 27.3 | 25.1 | 29.5 | 2016 | 25.8 | 24.2 | 27.4 |
| **Zambia** | 1996 | 28.1 | 26.0 | 30.1 | 2013 | 28.3 | 26.7 | 29.9 |
| **Zimbabwe** | 1994 | 23.7 | 21.3 | 26.0 | 2015 | 28.5 | 26.1 | 31.0 |
|  |  |  |  |  |  |  |  |  |
| **Country** | **Year** | **Estimate <20 years** | **Lower CI** | **Upper CI** | **Year** | **Estimate <20 years** | **Lower CI** | **Upper CI** |
| **Ethiopia** | 2000 | 43.6 | 41.8 | 45.4 | 2016 | 38.4 | 35.7 | 42 |
| **Kenya** | 1993 | 52.1 | 49.7 | 54.6 | 2014 | 43.0 | 41.2 | 44.8 |
| **Madagascar** | 1997 | 56.6 | 53.9 | 59.2 | 2008 | 56.8 | 55.0 | 58.6 |
| **Malawi** | 1992 | 63.3 | 60.3 | 66.3 | 2015 | 60.5 | 58.7 | 62.2 |
| **Mozambique** | 1997 | 65.3 | 63.1 | 67.6 | 2011 | 68.0 | 66.1 | 69.8 |
| **Namibia** | 1992 | 41.6 | 38.6 | 44.5 | 2013 | 34.6 | 32.1 | 37.2 |
| **Rwanda** | 1992 | 24.6 | 22.3 | 27.0 | 2014 | 20.5 | 18.8 | 22.1 |
| **Tanzania** | 1996 | 52.4 | 50.0 | 54.8 | 2015 | 49.8 | 47.4 | 52.2 |
| **Uganda** | 1995 | 66.4 | 64.1 | 68.7 | 2016 | 54.1 | 52.3 | 56.0 |
| **Zambia** | 1996 | 63.3 | 61.1 | 65.5 | 2013 | 58.9 | 57.2 | 60.7 |
| **Zimbabwe** | 1994 | 46.9 | 44.2 | 49.7 | 2015 | 50.5 | 47.8 | 53.2 |
|  |  |  |  |  |  |  |  |  |
| **Country** | **Year** | **Estimate <16 years** | **Lower CI** | **Upper CI** | **Year** | **Estimate <16 years** | **Lower CI** | **Upper CI** |
| **Benin** | 1996 | 5.9 | 4.5 | 7.4 | 2011 | 9.9 | 8.8 | 11.1 |
| **Burkina Faso** | 1993 | 9.2 | 7.6 | 10.8 | 2010 | 6.9 | 6.0 | 7.8 |
| **Cameroon** | 1991 | 21.2 | 18.3 | 24.0 | 2011 | 12.0 | 10.9 | 13.1 |
| **Chad** | 1996 | 19.7 | 17.5 | 21.9 | 2014 | 26.7 | 24.8 | 28.6 |
| **Cote d'Ivoire** | 1994 | 18.2 | 16.3 | 20.0 | 2011 | 12.0 | 10.6 | 13.4 |
| **Ghana** | 1993 | 6.5 | 4.8 | 8.2 | 2014 | 5.1 | 3.8 | 6.3 |
| **Guinea** | 1999 | 22.9 | 20.5 | 25.4 | 2012 | 19.6 | 17.6 | 21.5 |
| **Mali** | 1995 | 16.0 | 14.2 | 17.8 | 2012 | 22.3 | 20.4 | 24.2 |
| **Niger** | 1998 | 19.1 | 17.0 | 21.1 | 2012 | 21.9 | 20.0 | 23.7 |
| **Nigeria** | 1990 | 18.7 | 16.9 | 20.6 | 2013 | 11.7 | 10.4 | 13.0 |
| **Senegal** | 1997 | 9.7 | 7.8 | 11.6 | 2017 | 5.8 | 5.0 | 6.7 |
|  |  |  |  |  |  |  |  |  |
| **Country** | **Year** | **Estimate 16/17 years** | **Lower CI** | **Upper CI** | **Year** | **Estimate 16 / 17 years** | **Lower CI** | **Upper CI** |
| **Benin** | 1996 | 17.5 | 15.2 | 19.8 | 2011 | 13.4 | 12.1 | 14.6 |
| **Burkina Faso** | 1993 | 22.3 | 20.0 | 24.7 | 2010 | 21.3 | 19.9 | 22.7 |
| **Cameroon** | 1991 | 25.1 | 22.0 | 28.1 | 2011 | 17.9 | 16.5 | 19.2 |
| **Chad** | 1996 | 25.7 | 23.3 | 28.1 | 2014 | 23.9 | 22.1 | 25.8 |
| **Cote d'Ivoire** | 1994 | 25.5 | 23.3 | 27.6 | 2011 | 19.1 | 17.4 | 20.8 |
| **Ghana** | 1993 | 18.0 | 15.4 | 20.6 | 2008 | 11.8 | 9.9 | 13.8 |
| **Guinea** | 1999 | 24.3 | 21.8 | 26.9 | 2012 | 20.5 | 18.5 | 22.4 |
| **Mali** | 1995 | 30.0 | 27.8 | 32.3 | 2012 | 23.9 | 22.0 | 25.9 |
| **Niger** | 1998 | 27.4 | 25.1 | 29.8 | 2012 | 26.3 | 24.4 | 28.3 |
| **Nigeria** | 1990 | 16.2 | 14.5 | 18.0 | 2013 | 17.4 | 15.9 | 18.8 |
| **Senegal** | 1997 | 16.1 | 13.9 | 18.4 | 2017 | 10.5 | 9.2 | 11.8 |
|  |  |  |  |  |  |  |  |  |
| **Country** | **Year** | **Estimate 18/19 years** | **Lower CI** | **Upper CI** | **Year** | **Estimate 18/19 years** | **Lower CI** | **Upper CI** |
| **Benin** | 1996 | 26.5 | 23.7 | 29.2 | 2011 | 18.3 | 16.8 | 19.7 |
| **Burkina Faso** | 1993 | 30.9 | 28.3 | 33.5 | 2010 | 29.1 | 27.5 | 30.7 |
| **Cameroon** | 1991 | 20.6 | 17.8 | 23.4 | 2011 | 19.5 | 18.1 | 20.9 |
| **Chad** | 1996 | 25.6 | 23.2 | 28.1 | 2014 | 19.3 | 17.6 | 21.1 |
| **Cote d'Ivoire** | 1994 | 19.6 | 17.7 | 21.5 | 2011 | 19.1 | 17.4 | 21.1 |
| **Ghana** | 1993 | 24.0 | 21.1 | 26.9 | 2014 | 14.7 | 12.6 | 16.8 |
| **Guinea** | 1999 | 18.9 | 16.6 | 21.2 | 2012 | 19.6 | 13.9 | 18.9 |
| **Mali** | 1995 | 23.8 | 21.7 | 25.9 | 2012 | 22.0 | 19.9 | 24.2 |
| **Niger** | 1998 | 23.7 | 21.5 | 26.0 | 2012 | 25.6 | 23.3 | 28.0 |
| **Nigeria** | 1990 | 18.6 | 16.8 | 20.5 | 2013 | 17.7 | 16.4 | 18.9 |
| **Senegal** | 1997 | 17.2 | 15.1 | 19.4 | 2017 | 16.4 | 14.4 | 18.3 |
|  |  |  |  |  |  |  |  |  |
| **Country** | **Year** | **Estimate <20 years** | **Lower CI** | **Upper CI** | **Year** | **Estimate <20 years** | **Lower CI** | **Upper CI** |
| **Benin** | 1996 | 49.9 | 46.8 | 52.9 | 2011 | 41.6 | 39.8 | 43.4 |
| **Burkina Faso** | 1993 | 62.4 | 59.7 | 65.1 | 2010 | 57.3 | 55.6 | 59.0 |
| **Cameroon** | 1991 | 66.8 | 63.5 | 70.1 | 2011 | 49.4 | 47.6 | 51.1 |
| **Chad** | 1996 | 71.0 | 68.5 | 73.5 | 2014 | 69.9 | 67.9 | 72.0 |
| **Cote d'Ivoire** | 1994 | 63.2 | 60.9 | 65.6 | 2011 | 50.2 | 48.0 | 52.4 |
| **Ghana** | 1993 | 43.1 | 40.7 | 45.5 | 2014 | 31.6 | 28.8 | 34.4 |
| **Guinea** | 1999 | 66.2 | 63.3 | 69.4 | 2012 | 59.6 | 57.2 | 62.0 |
| **Mali** | 1995 | 66.8 | 63.4 | 69.0 | 2012 | 68.2 | 66.1 | 70.3 |
| **Niger** | 1998 | 69.8 | 67.6 | 72.1 | 2012 | 73.9 | 71.9 | 75.8 |
| **Nigeria** | 1990 | 70.2 | 67.8 | 72.7 | 2013 | 46.7 | 45.5 | 47.9 |
| **Senegal** | 1997 | 53.6 | 51.2 | 55.9 | 2014 | 32.5 | 30.5 | 34.4 |
